# Regulation of metabolism by long, non-coding RNAs

**DOI:** 10.3389/fgene.2014.00057

**Published:** 2014-03-25

**Authors:** Jan-Wilhelm Kornfeld, Jens C. Brüning

**Affiliations:** ^1^Cologne Excellence Cluster on Cellular Stress Responses in Aging Associated DiseasesKöln, Germany; ^2^Max-Planck-Institute for Neurological ResearchKöln, Germany; ^3^Department of Mouse Genetics and Metabolism and Center for Molecular Medicine Cologne, Institute for Genetics at the University Hospital of Cologne, University of CologneCologne, Germany; ^4^Center for Endocrinology, Diabetes and Preventive Medicine, University Hospital Cologne, University of Cologne, CologneGermany

**Keywords:** lncRNAs, glucose homeostasis, metabolism and obesity, non-coding RNA (ncRNA), cell differentiation

## Abstract

Our understanding of genomic regulation was revolutionized by the discovery that the genome is pervasively transcribed, giving rise to thousands of mostly uncharacterized non-coding ribonucleic acids (ncRNAs). Long, ncRNAs (lncRNAs) have thus emerged as a novel class of functional RNAs that impinge on gene regulation by a broad spectrum of mechanisms such as the recruitment of epigenetic modifier proteins, control of mRNA decay and DNA sequestration of transcription factors. We review those lncRNAs that are implicated in differentiation and homeostasis of metabolic tissues and present novel concepts on how lncRNAs might act on energy and glucose homeostasis. Finally, the control of circadian rhythm by lncRNAs is an emerging principles of lncRNA-mediated gene regulation.

## INTRODUCTION

### THE NON-CODING GENOME

**FIGURE 1:**
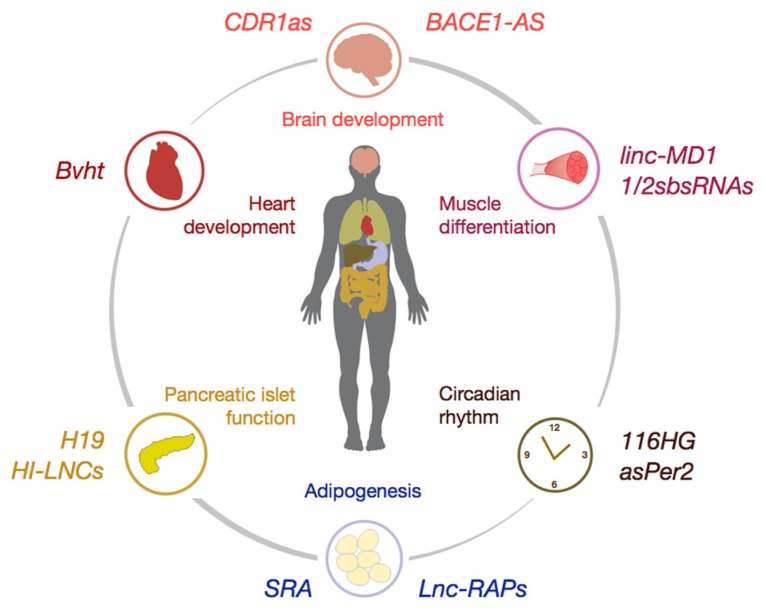
**Schematic illustration of selected lncRNAs involved in the control of organ differentiation and development (e.g., *CDR1as*, *Bvht*, and* linc-MD1*), tissue homeostasis (e.g., *Lnc-RAPs* and *HI-LNCs*) and control of circadian rhythm (e.g., *asPer2*).** For detailed description of lncRNA mode-of-action please refer to the main text.

The canonical view of mammalian genomes revolves around the notion that the roughly 20,000 proteins within mammalian genomes are interspersed by somewhat conserved, yet functionally redundant non-coding regions with only limited regulatory potential. Regulatory properties of these “non-coding” regions were only attributed to *cis*-regulatory elements such as promoters or *cis/trans*-enhancer regions. This paradigm was fundamentally called into question by results obtained from whole-transcriptome sequencing efforts [e.g., by the ENCODE consortium (Birney et al., 2007; Thomas et al., 2007)] over the last decade that have revealed the pervasive transcription of mammalian genomes (Carninci et al., 2005; Birney et al., 2007; Derrien et al., 2012). Although the magnitude of pervasiveness remains under debate (van Bakel et al., 2010; Clark et al., 2011), recent meta-analyses of human ribonucleic acid-sequencing (RNA-Seq) datasets have confirmed that >80% of genomic sequences are rediscovered within RNA transcripts, often in a temporally and spatially specific manner (Hangauer et al., 2013). One logical consequence of pervasive transcription is the abundance of non-coding RNAs (ncRNAs) within mammalian genomes, a phenomenon which holds true for most eukaryotic species ranging from yeast (David et al., 2006), to *Drosophila* (Stolc et al., 2004), plants (Li et al., 2006) and humans (Hangauer et al., 2013). Given the predicted high number of ncRNAs within mammalian genomes, which probably surpasses that of coding genes, it is not surprising that a large conceptual void remains about the multifaceted role of ncRNAs in regulation of gene expression. Researchers have historically divided ncRNAs into small ncRNAs (sRNAs <200 nt length) such as microRNAs (miRNAs) and small nucleolar RNAs (snoRNAs) in contrast to so-termed long ncRNAs (lncRNAs; >200 nt length). Until today, the identification of biological processes, which are regulated by miRNAs as well as the elucidation of mRNA targets, which are posttranscriptionally regulated by disease-associated miRNAs remains an important focus of research (Bartel, 2009; Carthew and Sontheimer, 2009). It was demonstrated that the spectrum of biological processes, which are regulated by miRNAs, ranges from the development of organs, the homeostatic regulation of cellular metabolism to aging and neurodegenerative disorders. Although miRNAs are central to the understanding of the non-coding genome, the regulation of energy homeostasis and metabolism by miRNAs has been meticulously reviewed elsewhere (Lynn, 2009; Rottiers and Naar, 2012; Kim and Kyung Lee, 2013) and goes beyond the scope of this review. In contrast to miRNAs, the role of lncRNAs in control of metabolism and energy homeostasis remains rather elusive. Thus, we here review the known roles for lncRNAs, which probably constitute the numerical majority of ncRNAs encoded within mammalian genomes, during differentiation, homeostasis and metabolic regulation of tissues (**Figure 1**).

### LONG NON-CODING RNAs

Those lncRNAs that were initially discovered in the late eighties had distinct, at that time considered exotic functions such as X chromosome inactivation in females by the lncRNA *XIST* (Penny et al., 1996). Another historical example was the imprinted lncRNA *H19*, which is involved in repression of *Igf2* (Pachnis et al., 1988). After the discovery of pervasive genomic transcription it became clear that lncRNAs do not represent an exotic observation, but rather a prominent feature of the genome (Birney et al., 2007). Although the number of lncRNAs is still debated, recent meta-analyses posit the human genome to give rise to >60,000 lncRNA, albeit the majority is probably expressed at low levels (Derrien et al., 2012; Hangauer et al., 2013; for current lncRNA numbers consult NONCODE Version 4, www.noncode.org). Interestingly, lncRNAs on one hand exhibit many similarities with protein-coding transcripts: As true for mRNAs, lncRNAs are transcribed by RNA-polymerase (Pol) II (Guttman et al., 2009), spliced at canonical splicing sites (Chew et al., 2013), are partly polyadenylated (Cabili et al., 2011) and even associate with polysomes (Guttman et al., 2013). Further, lncRNAs harbor the same chromatin marks of H3K4 and H3K36 trimethylation as found in active promoters and transcribed regions of protein-coding transcripts, respectively, a phenomenon which aided in the identification of novel lncRNAs (Mikkelsen et al., 2007). It is noteworthy that the notion of distinct mRNA-like trimethylation marks on actively transcribed lncRNAs is incompatible with the criticism brought forward according to which lncRNAs are merely generated by unspecific Pol II activity which leads to low-level transcription of non-coding sequences (“transcriptional noise”; Derrien et al., 2012). On the other hand, certain features set lncRNAs apart from protein-coding genes: Generally, lncRNAs are expressed at lower levels, are less evolutionarily conserved and less frequently associate with ribosomes than protein-coding transcripts (Hangauer et al., 2013). Further, lncRNA are shorter than coding genes and are composed of a unique gene structure of usually 1–2 exons. Of note, some lncRNAs do give rise to small peptides and may act as both, coding and non-coding, transcript (reviewed here Dinger et al., 2008).

## PRINCIPLES OF lncRNA-MEDIATED GENE REGULATION

Currently, intensive research efforts are underway to better understand the molecular basis of gene regulation by lncRNAs. To date, four major paradigms have emerged on how lncRNA impinge on gene regulation:

### (EPIGENETIC) REGULATION OF GENE TRANSCRIPTION

LncRNAs are able to bring gene-regulatory DNA-binding proteins and DNA sequences into close proximity and thus constitute an ideal docking platform for recruiting epigenetic modifiers to distinct genomic loci in *cis*- or *trans*. Indeed, early insights into lncRNA-based gene regulation have revealed the recruitment of the inhibitory polycomb repressive complex (PRC) 2 and the activating Trithrorax/MLL chromatin modifiers to specific genomic loci by the lncRNAs *HOTAIR *(Rinn et al., 2007) and *HOTTIP *(Wang et al., 2011), respectively. PRC2 and MLL then mark distinct lysine residues within histones via trimethylation, leading to inhibition or activation of gene transcription. In a similar fashion, the lncRNA *ANRIL *silences the *INK4a *tumor suppressor allele by H3K27 trimethylation via recruiting the Polycomb chromatin modifier CBX7 (Yap et al., 2010). The percentage of lncRNAs implicated in (epigenetic) gene regulation was systematically quantified by interrogating the PRC2 interactome using chromatin-state maps. This revealed the abundant interaction of Polycomb repressor proteins with up to 20% of expressed lncRNAs (Khalil et al., 2009). Thus, one prominent role of lncRNAs relates to writing and erasing chromatin marks, thereby controlling the epigenetic state of lncRNA-bound genomic loci (Spitale et al., 2011). In a systematic attempt to interrogate the function of 3019 human lncRNAs, Orom et al. (2010) revealed that a significant portion of the lncRNome possesses *cis*-regulatory enhancer properties (hitherto termed enhancer-like RNAs, eRNAs), which control the expression of neighboring protein-coding genes. Elegant follow-up studies using chromosome conformation capture (3C) technology revealed that the co-activator complex Mediator is involved in tethering eRNAs to their gene targets. Hence, lncRNAs regulate the three-dimensional (3D) structure of chromosomes via Mediator-dependent chromosome looping (Lai et al., 2013), thereby bridging large intra- and interchromosomal distances in order to activate distal promoters (reviewed in Orom and Shiekhattar, 2013). This study nicely complemented reports about the lncRNA-mediated regulation of *HOXA* genes, in which chromosomal looping brings the eRNA *HOTTIP* in proximity to its target genes, marks the chromatin by H3K4 trimethylation and thus activates gene transcription (Wang et al., 2011). Taken together, the translation of the information content that lies within higher-order (3D) structures of chromosomes into (epigenetic) modifications of chromatin and regulation of gene transcription seems to be an emerging principle of lncRNA function.

### PROCESSING/DEGRADATION OF mRNA

Every step of RNA metabolism is subjected to fine-tuned and complex regulation (reviewed in Moore, 2005). LncRNAs have recently been involved in the control of RNA stability, the processing of (pre)-mRNAs and the regulation of mRNA decay. Natural antisense transcripts (NATs) are lncRNAs which are characterized by their location antisense to other coding or non-coding transcripts (Faghihi and Wahlestedt, 2009). The upregulation of NATs often causes downregulation of protein-coding transcripts on the opposite strand by the formation of RNA duplexes and triggering of cellular RNAi, although recently the NAT-mediated upregulation of protein-coding transcripts on the opposite strand were reported (Carrieri et al., 2012). The repressive effect of NATs onto the opposite strand not only holds true for duplexes consisting of (i) a protein-coding mRNA and a non-coding lncRNA NAT, but also for (ii) duplexes between a lncRNA NAT and another lncRNAs as demonstrated for lncRNAs which base-pair with and target the *PTEN* pseudogene *PTENpg1 *(Johnsson et al., 2013). The lncRNA-mediated regulation of (pre)-mRNA processing was demonstrated for the nuclear retained lncRNA *MALAT1*, which modulates alternative splicing via assembly of serine/arginine splicing factors within subnuclear compartments called nuclear speckles (Tripathi et al., 2010). Finally, the timely degradation of mRNAs by a process called Staufen-mediated decay (SMD) involves lncRNA-recipient sequences in the 3′UTR of SMD target genes. Here, intermolecular base-pairing between lncRNAs and sequences within the 3′UTR of Staufen (Stau) target genes triggers a cellular SMD response and the ensuing degradation of the transcript (Gong and Maquat, 2011; Wang et al., 2013). The *in vivo *significance of lncRNA-mediated SMD decay was underscored by the observation that epidermal differentiation critically depends on lncRNA-elicited mRNA decay. Here, a lncRNA called *TINCR* is recruited to specific sequences called “TINCR box” within *TINCR *target genes and elicits the decay of TINCR-bound transcripts by SMD (Kretz et al., 2013; Kretz, 2013).

### POSTTRANSCRIPTIONAL GENE REGULATION

MiRNAs recognize their gene targets by binding to 6–8 nt sequences called “seeds” located in the 3′UTR region of protein-coding RNAs (Bartel, 2009). It was demonstrated that recognition, binding and degradation/translational inactivation of miRNA targets is not necessarily confined to protein-coding transcripts. A so-called “competing endogenous RNA (ceRNA) hypothesis” was brought forward according to which protein-coding RNAs, miRNAs, and lncRNAs transcripts form large-scale regulatory networks which impinge on the expression of other transcripts independently of protein translation via competing for a limited pool of miRNAs (Salmena et al., 2011). Here, transcripts, so-called ceRNAs regulate the expression of other transcripts based on the similarity of their 3′UTR miRNA response elements (MRE) profile. According to this notion, two transcripts with a strong degree of common MREs can crosstalk to each other by competing for a given pool of miRNAs. Upregulation of one ceRNA increasingly “sponges” a limited pool of miRNAs and relieves the miRNA-mediated repressive tone on ceRNA-linked transcripts. The experimental confirmation of a ceRNA-like interdependency of protein-coding transcripts was first demonstrated for the tumor suppressor gene *PTEN* (Karreth et al., 2011; Tay et al., 2011), which “crosstalks” to hitherto unknown tumor suppressors. PTEN loss-of-function during cancerogenesis is also controlled by the genomic loss of its (non-coding) pseudogene *PTENP1* which acts as a ceRNA (Poliseno et al., 2010). An additional layer of posttranscriptional regulation by lncRNAs accordingly lies within the specific pattern of MREs within lncRNAs which allow it to influence the expression of coding and non-coding transcripts in a ceRNA-like fashion. This is exemplified by the upregulation of a non-coding antisense homologue of the beta-secretase BACE1 (*BACE1-AS*) that acts as BACE1 ceRNA and concomitantly increases Bace1 mRNA stability and leads to augmented deposition of Aβ-plaques in Alzheimer’s disease (AD; Faghihi et al., 2008, 2010). Finally, a novel, intriguing class of functional lncRNAs, which is encoded in eukaryotic genomes, is constituted by circular RNAs (circRNAs). CircRNAs are expressed at high levels, can act as ceRNAs and effectively sponge miRNAs as shown for the neuroendocrine miRNA miR-7 (Hansen et al., 2013; Memczak et al., 2013).

### REGULATION OF PROTEIN ACTIVITY

Ribonucleic acid possesses the unique biochemical property to recognize and bind most biomolecules including proteins with unprecedented affinity (Stoltenburg et al., 2007). Thus, lncRNAs can specifically bind proteins and elegant studies have attributed novel roles for lncRNAs in the control of tissue homeostasis via direct binding and modification of protein activity. For example, a lncRNA termed *Evi2 *was shown to form stable complexes with members of the Dlx/Dll family of transcription factors, which are crucial regulators of developmental timing in vertebrates, and thereby regulate their transcriptional output (Feng et al., 2006). Further, two lncRNAs termed *PRNBR1* and *PCGEM1* that are upregulated in aggressive prostate cancer, synergistically and coordinately bind the carboxyterminal part of the androgen receptor (AR) and are required for AR-dependent gene transcription. In androgen-refractory prostate cancer, *PRNBR1* and *PCGEM1* are robustly expressed and are implicated in the ligand-independent activation of AR signaling [AR “resistant” prostate cancer (Yang et al., 2013)]. Another emerging paradigm of lncRNA-mediated regulation of protein activity is the sequestration of transcription factors as exemplified by the lncRNA *Gas5*, which is induced under conditions of nutrient deprivation and cellular stress. *Gas5* acts as glucocorticoid receptor (GR) decoy by competing with GR-responsive elements (GREs) in gene promoters for binding to the DNA-binding domain of the GR (Kino et al., 2010). Increased levels of *Gas5* thus interfere with GR binding to the DNA and effectively inhibit transactivation of GR-dependent gene promoters. Another example is nuclear factor kappa b (NFkB) signaling, which translates extracellular, proinflammatory cues [e.g., by tumor necrosis factor alpha (TNF-α) receptor activation] into changes in gene expression. NFkB activation induces the transcription of a specific subset of lncRNAs, apart from the induction of classical inflammatory protein-coding genes. Among this subset of TNF-regulated lncRNAs, a lncRNA termed *Lethe* is recruited to the NFkB effector subunit RelA in an inducible fashion and inhibits RelA from DNA-binding and target gene activation (Rapicavoli et al., 2013). Finally, the hypoxia-regulated lncRNA *linc-p21* was shown to physically interact with hypoxia-inducible factor (HIF) 1alpha transcription factors. This HIF1a-*linc-p21 *circuit controls the hypoxia-evoked increases of the glycolytic “Warburg effect” in tumor cells (Yang et al., 2014).

## LncRNAs IN CONTROL OF METABOLISM

The regulation of metabolism and glucose homeostasis is orchestrated and fine-tuned by a complex interplay of tissues/organs. Currently, we are faced with an unprecedented rise of obesity in the civilized world and the concurrent increase in obesity-associated diseases such as insulin resistance and type 2 diabetes mellitus (T2D). Key to the understanding of whole-body metabolism are the pleiotropic effects of the anabolic master regulator insulin which simultaneously controls peripheral as well as central-nervous system-related aspects of metabolism (Kahn et al., 2006). Resistance toward the effects of insulin constitutes a key step in the development of metabolic disease. The exciting observation that insulin and insulin-like growth factor (IGF) 1 signaling also triggers distinct changes in lncRNA expression [e.g., of the lncRNA *CRNDE *(Ellis et al., 2013)] points to the fact that lncRNAs may also be implicated in the metabolic effects of insulin and the development of insulin resistance. Thus, a strong interest lies within the identification of lncRNA-mediated mechanisms governing energy and glucose homeostasis at the cell-intrinsic, organ and whole-body level.

## TISSUE-SPECIFIC REGULATION OF METABOLISM BY lncRNAs

### MAINTENANCE OF PANCREATIC BETA CELL IDENTITY

The main function of pancreatic islets lies within the synthesis, storage and secretion of insulin and glucagon, two hormonal regulators of glucose homeostasis. The possible control of islet development and function by lncRNAs was first demonstrated in studies which reported that the lncRNA *H19* is involved in the intergenerational transmission of diabetes mellitus [gestational diabetes mellitus (GDM)] and the GDM-associated impairments of islet infrastructure and function (Ding et al., 2012). Global lncRNA screening approaches conducted by Moran et al. (2012) systematically interrogated the lncRNA transcriptome in human pancreatic beta cells. Here, the dynamic, strand and tissue-specific regulation of >1,000 lncRNA was reported using integrated transcriptional and chromosomal maps. Utilizing RNA-Seq data of 16 non-pancreatic tissues, the aforementioned gene set of pancreatic lncRNAs was shown to be significantly more specific for islet cells (40–55% for intergenic and antisense lncRNAs, respectively) than protein-coding genes (9.4%). Furthermore, the upregulation of islet-specific lncRNAs during progenitor commitment, glucose-stimulated upregulation and the striking dysregulation of islet-specific lncRNAs in patients with T2D pointed to a pathophysiological role of lncRNAs in the homeostasis of pancreatic tissues. The fact that a significant percentage of mouse and human lncRNA orthologs display similar cell- and stage-specific expression patterns suggests that evolutionarily conserved properties of lncRNAs extend beyond their primary sequence. This study was corroborated by a publication from the McManus laboratory, which presented a new catalog of the human beta cell (non-coding) lncRNA transcriptome in which >1,000 lncRNA were expressed in an islet-specific fashion involving islet-specific splicing events and promoter utilization (Ku et al., 2012). However, the elucidation of the molecular mechanisms underlying lncRNA-mediated regulation of beta cell differentiation and function still await discovery.

### REGULATION OF ADIPOGENESIS AND ADIPOSE TISSUE PLASTICITY

The body harbors two principal types of adipose tissues which possess key functions in regulating the equilibrium between nutrient deposition and energy expenditure: Whereas white adipose tissue (WAT) serves as storage organ for excess nutrients, brown adipose tissue (BAT) dissipates the proton gradient across mitochondrial membranes to generate heat via the BAT-intrinsic uncoupling protein 1 (UCP1; Bartelt and Heeren, 2012). The accumulation of excess lipids that leads to low-grade inflammation in WAT has been linked to the development of insulin resistance in obese patients (Saltiel and Kahn, 2001; Gregor and Hotamisligil, 2011; Glass and Olefsky, 2012). Also, impaired BAT thermogenesis can contribute to the development of insulin resistance and obesity (Connolly et al., 1982; Feldmann et al., 2009). The fact that lncRNAs are implicated in the differentiation of adipose tissues (adipogenesis) is exemplified by the lncRNA *SRA*, which is required for full transactivation of the proadipogenic transcription factor Peroxisome proliferator-associated receptor gamma (Pparg). Concomitantly, RNAi-mediated *SRA* loss-of-function interfered with *in vitro* differentiation of 3T3-L1 preadipocytes (Xu et al., 2010). In a seminal study by Sun et al. (2013), the systematic implication of lncRNAs during adipogenesis was addressed. Using global transcriptome profiling of undifferentiated and mature adipocytes from the WAT and BAT lineages, the significant and specific regulation of 175 lncRNAs during adipogenesis was reported (Sun et al., 2013) of which a significant portion were enriched within adipose tissues. Finally, subsets of newly identified lncRNAs termed lncRAPs (lncRNAs Regulated in AdiPogenesis) were depleted *in vitro *using siRNAs. Distinct lncRAPs, which were specifically upregulated during adipogenesis and were induced by the proadipogenic transcription factors Cebpa and Pparg, were required for timely and complete maturation of adipocyte progenitor cells. These studies provide first evidence for a crucial role of lncRNAs in the control of adipogenesis and fat cell metabolism.

### DIFFERENTIATION OF SKELETAL MUSCLE AND CARDIOMYOCYTES

The differentiation of skeletal muscle cells (myogenesis) is regulated by a complex, yet well understood, evolutionarily conserved circuitry of protein-coding genes which control the timely growth, morphogenesis, and terminal maturation of muscle progenitors (myoblasts; Buckingham and Vincent, 2009). Here, the implication of noncoding RNAs was first shown via the contribution of myogenic miRNAs (myomiRs like miR-1 and miR-133) during myoblast commitment (Chen et al., 2006; Liu et al., 2008). Gong and Maquat (2011) reported that in human cells the degradation of distinct, nascent coding transcripts by Staufen-mediated decay (SMD) was regulated by lncRNAs. Here, the intermolecular base-pairing between *Alu *elements located within the 3′UTR of an SMD target and an *Alu *site localized within a class of lncRNAs called *1/2sbsRNAs* (1/2-STAU1-binding site RNAs) triggered SMD (Gong and Maquat, 2011). This process was interestingly conserved in rodents that lack canonical *Alu *repeats. Here, the mouse homologue of *1/2sbsRNA* was shown to be implicated in terminal differentiation of myoblast cells (Wang et al., 2013), indicating a function of lncRNAs in myogenesis. Another lncRNA termed *linc-MD1* is also critical for myogenesis. Here, increased levels of *linc-*MD1 trigger the muscle differentiation program by acting as a natural decoy for myomiRs miR-133 and miR-135 (Cesana et al., 2011). MiR-133 and -135 in turn repress the expression of two pro-myogenic transcription factors, MAML1 and MEF2C. Recent reports also revealed that *linc-MD1 *takes part in a molecular feedforward circuit involving the promyogenic protein HuR (Legnini et al., 2014). Collectively, *linc-MD1 *promotes terminal differentiation of myoblasts via acting as ceRNA for myogenic transcriptional regulators by sequestering anti-myogenic miRNAs. Interestingly, this complex ceRNA-based interplay of classical mRNAs, lncRNAs, and miRNAs was dysregulated in patients suffering from Duchenne muscular dystrophy (DMD), a condition of reduced terminal differentiation of myoblasts. Reinstating DMD-associated downregulation of *linc-MD1* expression via lentiviral delivery led to improved maturation of DMD myoblasts. In a study published by Klattenhoff et al. (2013), the heart-intrinsic lncRNA *Braveheart *(*Bvht*) was demonstrated to be required for differentiation of mesodermal progenitors toward mature cardiomyocytes via interaction with PRC2 epigenetic modifiers. This report for the first time implicated a tissue-specific lncRNA in maintaining cell fate during mammalian organogenesis.

### REGULATION OF NEUROGENESIS BY lncRNAs

The discovery that peripherally secreted hormones such as insulin and leptin control energy homeostasis and glucose metabolism via CNS-acting neurocircuits expanded our understanding on how the body ingests, stores and dissipates energy (Belgardt and Bruning, 2010). In an approach to identify lncRNAs, which are implicated in brain development and neurogenesis, Aprea et al. (2013) utilized transgenic *in vivo* approaches to isolate neural stem cells, partially committed neuronal precursor cells as well as terminally differentiated neurons and quantified the expression of lncRNAs. Several lncRNAs were identified that were involved in neurogenesis, neuroblast commitment and neuron survival as shown for the neuroregulatory lncRNA *Miat*. Thus, maintenance of the neuron stem cell pool and terminal differentiation of neuron progenitors are also under lncRNA-mediated control. This will hopefully entail studies in the future specifically addressing the regulation of defined neuronal circuits, which regulate peripheral metabolic by lncRNAs.

### REGULATION OF CIRCADIAN RHYTHM BY lncRNAs

The mammalian clock plays a fundamental role in the regulation of energy and glucose homeostasis. Dysregulation of the circadian rhythm underlies several metabolic pathologies like the development of insulin resistance and the metabolic syndrome (Marcheva et al., 2010; Hatori et al., 2013). In addition to the central clock located in the suprachiasmatic nucleus (SCN) of the pineal gland in the CNS, subordinate, tissue-specific clocks exist which are also key for the regulation of diurnal aspects of lipid metabolism, oscillations in core body temperature and timely insulin secretion from pancreatic beta cells (Cretenet et al., 2010; Marcheva et al., 2010; Gerhart-Hines et al., 2013). Interestingly, as found for plants (Hazen et al., 2009), lncRNAs are involved in the regulation of vertebrate circadian systems. A study published by Coon et al. showed that 112 lncRNAs are differentially expressed between day/night within the pineal gland of rats (Coon et al., 2012). An in-depth investigation of eight highly rhythmic lncRNA revealed the pivotal role of neuronal projections from the SCN as well as external zeitgebers like light exposure onto periodicity and amplitude of circadian lncRNAs. In addition, a report from Vollmers et al. (2012) observed that rhythmic expression of ncRNAs like NATs, lncRNAs and miRNAs leads to rhythmic chromatin modifications in the liver. Noteworthy, the circadian oscillator component Per2 itself is controlled by an antisense lncRNA termed *asPer2*. Reports about the brain-derived regulation of circadian metabolism remain scarce yet the pathogenesis of Prader–Willi syndrome (PWS), a CNS-controlled genetic disorder circadian rhythm with an associated dysregulation of metabolism and the development of obesity, was shown to be influenced by a PWS-associated lncRNA called *116G. *After splicing, a lncRNA consisting of the remnants of *116G* (termed *116HG*) bound to the transcriptional activator RBBP5 and ensures a physiological circadian rhythm in the brain. Mice deficient for *116HG* exhibit metabolic disorders due to the dysregulation of diurnally expressed circadian genes like *Clock*, *Cry1*, and *Per2* in the CNS (Powell et al., 2013).

### EMERGING CONCEPT: INTERCELLULAR COMMUNICATION BY EXOSOMAL lncRNAs?

Exosomes are small vesicles generated by budding of the plasma membrane and constitute a specific vehicle for intercellular communication. Upon release from donor cells, exosomal surface motifs serve as “address codes” for binding and endocytosis on acceptor cells. Specific exosomal shuttling RNAs (esRNAs) such as miRNAs can be packaged into exosomes and released after binding to recipient cells, thus constituting a novel and intriguing way for ncRNAs to regulate systemic aspects of metabolism (Ramachandran and Palanisamy, 2012). Similarly, the intercellular transport of high-density lipoproteins (HDL)-bound miRNAs that are released by distinct donor cells influence the miRNA profile of acceptor cells and concomitantly alter the gene expression in HDL-recipient target tissues (Vickers et al., 2011). Of note, deep sequencing of human exosomes revealed that lncRNAs are localized within micro-vesicles and may emerge as novel means of cellular communication (Huang et al., 2013). Although experimental proof of concept is still lacking, the endocrine transfer of exosomal lncRNA might represent a novel facette relevant for lncRNA-mediated control of metabolism.

## THERAPEUTIC OPPORTUNITIES OF lncRNA INHIBITION

A high economic interest lies in the development of sequence-specific compounds for the inhibition of disease-associated ncRNAs. Short, chemically modified ribonucleic acid compounds like locked nucleic acids (LNAs) efficiently silence the expression of ncRNAs such as miRNAs and are generally well tolerated *in vivo* (Krutzfeldt et al., 2005; Esau et al., 2006; Elmen et al., 2008). These anti-RNA compounds were initially tested in mice (Krutzfeldt et al., 2005) and adopted to the non-human primate situation (Elmen et al., 2008) with unprecedented speed. This approach will hopefully be extended to disease-associated lncRNA in the near future. Most *in vivo *studies to date concentrated on disease-associated miRNAs, that were critically involved in the development of insulin resistance and the deterioration of metabolic health (Jordan et al., 2011; Trajkovski et al., 2011; Zhou et al., 2012; Kornfeld et al., 2013). In contrast, most insights concerning the metabolic functions of lncRNAs were inferred from *in vitro* studies. The rising numbers of lncRNA knockout models [exemplified by a recent report on 18 lncRNA loss-of-function mouse models (Sauvageau et al., 2013)] showcase that in order to convincingly assess, whether lncRNAs are implicated in the *in vivo* control of metabolism, further animal models for lncRNA loss- and gain-of-function are needed. This is of timely importance as systemic antisense oligonucleotide (ASO)-mediated inhibition of disease-associated lncRNAs (even in difficult to target organs like skeletal muscle) effectively improves degenerative diseases like myotonic dystrophy type 1 (DM1) in mice (Wheeler et al., 2012).

## Conflict of Interest Statement

The authors declare that the research was conducted in the absence of any commercial or financial relationships that could be construed as a potential conflict of interest.

## References

[B1] Aprea J. Prenninger S. Dori M. Ghosh T. Monasor L. S. Wessendorf E. (2013). Transcriptome sequencing during mouse brain development identifies long non-coding RNAs functionally involved in neurogenic commitment. *EMBO J.* 32 3145–3160 10.1038/emboj.2013.245 24240175 PMC3981144

[B2] Bartel D. P. (2009). MicroRNAs: target recognition and regulatory functions. *Cell* 136 215–233 10.1016/j.cell.2009.01.002 19167326 PMC3794896

[B3] Bartelt A. Heeren J. (2012). The holy grail of metabolic disease: brown adipose tissue. *Curr. Opin. Lipidol.* 23 190–195 10.1097/MOL.0b013e328352dcef 22449813

[B4] Belgardt B. F. Bruning J. C. (2010). CNS leptin and insulin action in the control of energy homeostasis. *Ann. N. Y. Acad. Sci.* 1212 97–113 10.1111/j.1749-6632.2010.05799.x 21070248

[B5] Birney E. Stamatoyannopoulos J. A. Dutta A. Guigó R. Gingeras T. R. Margulies E. H. (2007). Identification and analysis of functional elements in 1% of the human genome by the ENCODE pilot project. *Nature* 447 799–816 10.1038/nature05874 17571346 PMC2212820

[B6] Buckingham M. Vincent S. D. (2009). Distinct and dynamic myogenic populations in the vertebrate embryo. *Curr. Opin. Genet. Dev.* 19 444–453 10.1016/j.gde.2009.08.001 19762225

[B7] Cabili M. N. Trapnell C. Goff L. Koziol M. Tazon-Vega B. Regev A. (2011). Integrative annotation of human large intergenic noncoding RNAs reveals global properties and specific subclasses. *Genes Dev.* 25 1915–1927 10.1101/gad.17446611 21890647 PMC3185964

[B8] Carninci P. Kasukawa T. Katayama S. Gough J. Frith M. C. Maeda N. (2005). The transcriptional landscape of the mammalian genome. *Science* 309 1559–1563 10.1126/science.1112014 16141072

[B9] Carrieri C. Cimatti L. Biagioli M. Beugnet A. Zucchelli S. Fedele S. (2012). Long non-coding antisense RNA controls Uchl1 translation through an embedded SINEB2 repeat. *Nature* 491 454–457 10.1038/nature11508 23064229

[B10] Carthew R. W. Sontheimer E. J. (2009). Origins and Mechanisms of miRNAs and siRNAs. *Cell* 136 642–655 10.1016/j.cell.2009.01.035 19239886 PMC2675692

[B11] Cesana M. Cacchiarelli D. Legnini I. Santini T. Sthandier O. Chinappi M. (2011). A long noncoding RNA controls muscle differentiation by functioning as a competing endogenous RNA. *Cell* 147 358–369 10.1016/j.cell.2011.09.028 22000014 PMC3234495

[B12] Chen J. F. Mandel E. M. Thomson J. M. Wu Q. Callis T. E. Hammond S. M. (2006). The role of microRNA-1 and microRNA-133 in skeletal muscle proliferation and differentiation. *Nat. Genet.* 38 228–233 10.1038/ng1725 16380711 PMC2538576

[B13] Chew G. L. Pauli A. Rinn J. L. Regev A. Schier A. F. Valen E. (2013). Ribosome profiling reveals resemblance between long non-coding RNAs and 5′ leaders of coding RNAs. *Development* 140 2828–2834 10.1242/dev.098343 23698349 PMC3678345

[B14] Clark M. B. Amaral P. P. Schlesinger F. J. Dinger M. E. Taft R. J. Rinn J. L. (2011). The reality of pervasive transcription. *PLoS Biol.* 9:e1000625; discussion e1001102 10.1371/journal.pbio.1000625 PMC3134446 21765801

[B15] Connolly E. Morrisey R. D. Carnie J. A. (1982). The effect of interscapular brown adipose tissue removal on body-weight and cold response in the mouse. *Br. J. Nutr.* 47 653–658 10.1079/BJN19820077 6282304

[B16] Coon S. L. Munson P. J. Cherukuri P. F. Sugden D. Rath M. F. Moller M. (2012). Circadian changes in long noncoding RNAs in the pineal gland. *Proc. Natl. Acad. Sci. U. S. A.* 109 13319–13324 10.1073/pnas.1207748109 22864914 PMC3421215

[B17] Cretenet G. Le Clech M. Gachon F. (2010). Circadian clock-coordinated 12 Hr period rhythmic activation of the IRE1alpha pathway controls lipid metabolism in mouse liver. *Cell Metab.* 11 47–57 10.1016/j.cmet.2009.11.002 20074527

[B18] David L. Huber W. Granovskaia M. Toedling J. Palm C. J. Bofkin L. (2006). A high-resolution map of transcription in the yeast genome. *Proc. Natl. Acad. Sci. U.S.A.* 103 5320–5325 10.1073/pnas.0601091103 16569694 PMC1414796

[B19] Derrien T. Johnson R. Bussotti G. Tanzer A. Djebali S. Tilgner H. (2012). The GENCODE v7 catalog of human long noncoding RNAs: analysis of their gene structure, evolution, and expression. *Genome Res.* 22 1775–1789 10.1101/gr.132159.111 22955988 PMC3431493

[B20] Ding G. L. Wang F. F. Shu J. Tian S. Jiang Y. Zhang D. (2012). Transgenerational glucose intolerance with Igf2/H19 epigenetic alterations in mouse islet induced by intrauterine hyperglycemia. *Diabetes* 61 1133–1142 10.2337/db11-1314 22447856 PMC3331740

[B21] Dinger M. E. Pang K. C. Mercer T. R. Mattick J. S. (2008). Differentiating protein-coding and noncoding RNA: challenges and ambiguities. *PLoS Comput. Biol.* 4:e1000176 10.1371/journal.pcbi.1000176 PMC2518207 19043537

[B22] Ellis B. C. Graham L. D. Molloy P. L. (2013). CRNDE, a long non-coding RNA responsive to insulin/IGF signaling, regulates genes involved in central metabolism. *Biochim. Biophys. Acta* 1843 372–386 10.1016/j.bbamcr.2013.10.016 24184209

[B23] Elmen J. Lindow M. Schütz S. Lawrence M. Petri A. Obad S. (2008). LNA-mediated microRNA silencing in non-human primates. *Nature* 452 896–899 10.1038/nature06783 18368051

[B24] Esau C. Davis S. Murray S. F. Yu X. X. Pandey S. K. Pear M. (2006). miR-122 regulation of lipid metabolism revealed by *in vivo* antisense targeting. *Cell Metab.* 3 87–98 10.1016/j.cmet.2006.01.005 16459310

[B25] Faghihi M. A. Modarresi F. Khalil A. M. Wood D. E. Sahagan B. G. Morgan T. E. (2008). Expression of a noncoding RNA is elevated in Alzheimer's disease and drives rapid feed-forward regulation of beta-secretase. *Nat. Med.* 14 723–730 10.1038/nm1784 18587408 PMC2826895

[B26] Faghihi M. A. Wahlestedt C. (2009). Regulatory roles of natural antisense transcripts. *Nat. Rev. Mol. Cell Biol.* 10 637–643 10.1038/nrm2738 19638999 PMC2850559

[B27] Faghihi M. A. Zhang M. Huang J. Modarresi F. Van der Brug M. P. Nalls M. A. (2010). Evidence for natural antisense transcript-mediated inhibition of microRNA function. *Genome Biol.* 11 R56 10.1186/gb-2010-11-5-r56 PMC2898074 20507594

[B28] Feldmann H. M. Golozoubova V. Cannon B. Nedergaard J. (2009). UCP1 ablation induces obesity and abolishes diet-induced thermogenesis in mice exempt from thermal stress by living at thermoneutrality. *Cell Metab.* 9 203–209 10.1016/j.cmet.2008.12.014 19187776

[B29] Feng J. Bi C. Clark B. S. Mady R. Shah P. Kohtz J. D. (2006). The Evf-2 noncoding RNA is transcribed from the Dlx-5/6 ultraconserved region and functions as a Dlx-2 transcriptional coactivator. *Genes Dev.* 20 1470–1484 10.1101/gad.1416106 16705037 PMC1475760

[B30] Gerhart-Hines Z. Feng D. Emmett M. J. Everett L. J. Loro E. Briggs E. R. (2013). The nuclear receptor Rev-erbalpha controls circadian thermogenic plasticity. *Nature* 503 410–413 10.1038/nature12642 24162845 PMC3839416

[B31] Glass C. K. Olefsky J. M. (2012). Inflammation and lipid signaling in the etiology of insulin resistance. *Cell Metab.* 15 635–645 10.1016/j.cmet.2012.04.001 22560216 PMC4156155

[B32] Gong C. Maquat L. E. (2011). lncRNAs transactivate STAU1-mediated mRNA decay by duplexing with 3′ UTRs via Alu elements. *Nature* 470 284–288 10.1038/nature09701 21307942 PMC3073508

[B33] Gregor M. F. Hotamisligil G. S. (2011). Inflammatory mechanisms in obesity. *Annu. Rev. Immunol.* 29 415–445 10.1146/annurev-immunol-031210-101322 21219177

[B34] Guttman M. Amit I. Garber M. French C. Lin M. F. Feldser D. (2009). Chromatin signature reveals over a thousand highly conserved large non-coding RNAs in mammals. *Nature* 458 223–227 10.1038/nature07672 19182780 PMC2754849

[B35] Guttman M. Russell P. Ingolia N. T. Weissman J. S. Lander E. S. (2013). Ribosome profiling provides evidence that large noncoding RNAs do not encode proteins. *Cell* 154 240–251 10.1016/j.cell.2013.06.009 23810193 PMC3756563

[B36] Hangauer M. J. Vaughn I. W. McManus M. T. (2013). Pervasive transcription of the human genome produces thousands of previously unidentified long intergenic noncoding RNAs. *PLoS Genet.* 9:e1003569 10.1371/journal.pgen.1003569 PMC3688513 23818866

[B37] Hansen T. B. Jensen T. I. Clausen B. H. Bramsen J. B. Finsen B. Damgaard C. K. (2013). Natural RNA circles function as efficient microRNA sponges. *Nature* 495 384–388 10.1038/nature11993 23446346

[B38] Hatori M. Vollmers C. Zarrinpar A. DiTacchio L. Bushong E. A. Gill S. (2013). Time-restricted feeding without reducing caloric intake prevents metabolic diseases in mice fed a high-fat diet. *Cell Metab.* 15 848–860 10.1016/j.cmet.2012.04.019 22608008 PMC3491655

[B39] Hazen S. P. Naef F. Quisel T. Gendron J. M. Chen H. Ecker J. R. (2009). Exploring the transcriptional landscape of plant circadian rhythms using genome tiling arrays. *Genome Biol.* 10 R17 10.1186/gb-2009-10-2-r17 PMC2688271 19210792

[B40] Huang X. Yuan T. Tschannen M. Sun Z. Jacob H. Du M. (2013). Characterization of human plasma-derived exosomal RNAs by deep sequencing. *BMC Genomics* 14 319 10.1186/1471-2164-14-319 PMC3653748 23663360

[B41] Johnsson P. Ackley A. Vidarsdottir L. Lui W. O. Corcoran M. Grandér D. (2013). A pseudogene long-noncoding-RNA network regulates PTEN transcription and translation in human cells. *Nat. Struct. Mol. Biol.* 20 440–446 10.1038/nsmb.2516 23435381 PMC3618526

[B42] Jordan S. D. Krüger M. Willmes D. M. Redemann N. Wunderlich F. T. Brönneke H. S. (2011). Obesity-induced overexpression of miRNA-143 inhibits insulin-stimulated AKT activation and impairs glucose metabolism. *Nat. Cell Biol.* 13 434–446 10.1038/ncb2211 21441927

[B43] Kahn S. E. Hull R. L. Utzschneider K. M. (2006). Mechanisms linking obesity to insulin resistance and type 2 diabetes. *Nature* 444 840–846 10.1038/nature05482 17167471

[B44] Karreth F. A. Tay Y. Perna D. Ala U. Tan S. M. Rust A. G. (2011). *In vivo* identification of tumor- suppressive PTEN ceRNAs in an oncogenic BRAF-induced mouse model of melanoma. *Cell* 147 382–395 10.1016/j.cell.2011.09.032 22000016 PMC3236086

[B45] Khalil A. M. Guttman M. Huarte M. Garber M. Raj A. Rivea Morales D. (2009). Many human large intergenic noncoding RNAs associate with chromatin-modifying complexes and affect gene expression. *Proc. Nat. Acad. Sci. U.S.A.* 106 11667–11672 10.1073/pnas.0904715106 PMC2704857 19571010

[B46] Kim W Kyung Lee E. (2013). Post-transcriptional regulation in metabolic diseases. *RNA Biol.* 9 772–780 10.4161/rna.20091 22664919 PMC3495744

[B47] Kino T. Hurt D. E. Ichijo T. Nader N. Chrousos G. P. (2010). Noncoding RNA gas5 is a growth arrest- and starvation-associated repressor of the glucocorticoid receptor. *Sci. Signal.* 3 ra8 10.1126/scisignal.2000568 PMC2819218 20124551

[B48] Klattenhoff C. A. Scheuermann J. C. Surface L. E. Bradley R. K. Fields P. A. Steinhauser M. L. (2013). Braveheart, a long noncoding RNA required for cardiovascular lineage commitment. *Cell* 152 570–583 10.1016/j.cell.2013.01.003 23352431 PMC3563769

[B49] Kornfeld J. W. Baitzel C. Könner A. C. Nicholls H. T. Vogt M. C. Herrmanns K. (2013). Obesity-induced overexpression of miR-802 impairs glucose metabolism through silencing of Hnf1b. *Nature* 494 111–115 10.1038/nature11793 23389544

[B50] Kretz M. (2013). TINCR, staufen1, and cellular differentiation. *RNA Biol.* 10 1597–1601 10.4161/rna.26249 PMC3866239 24019000

[B51] Kretz M. Siprashvili Z. Chu C. Webster D. E. Zehnder A. Qu K. (2013). Control of somatic tissue differentiation by the long non-coding RNA TINCR. *Nature* 93 231–235 10.1038/nature11661 23201690 PMC3674581

[B52] Krutzfeldt J. Rajewsky N. Braich R. Rajeev K. G. Tuschl T. Manoharan M. (2005). Silencing of microRNAs *in vivo* with `antagomirs.' *Nature* 438 685–689 10.1038/nature04303 16258535

[B53] Ku G. M. Kim H. Vaughn I. W. Hangauer M. J. Myung Oh C. German M. S. (2012). Research resource: RNA-Seq reveals unique features of the pancreatic beta-cell transcriptome. *Mol. Endocrinol.* 26 1783–1792 10.1210/me.2012-1176 22915829 PMC3458219

[B54] Lai F. Orom U. A. Cesaroni M. Beringer M. Taatjes D. J. Blobel G. A. (2013). Activating RNAs associate with Mediator to enhance chromatin architecture and transcription. *Nature* 494 497–501 10.1038/nature11884 23417068 PMC4109059

[B55] Legnini I. Morlando M. Mangiavacchi A. Fatica A. Bozzoni I. A. (2014). Feedforward regulatory loop between HuR and the long noncoding RNA linc-MD1 controls early phases of myogenesis. *Mol. Cell* 53 506–514 10.1016/j.molcel.2013.12.012 24440503 PMC3919156

[B56] Li L. Wang X. Stolc V. Li X. Zhang D. Su N. (2006). Genome-wide transcription analyses in rice using tiling microarrays. *Nat. Genet.* 38 124–129 10.1038/ng1704 16369532

[B57] Liu N. Bezprozvannaya S. Williams A. H. Qi X. Richardson J. A. Bassel-Duby R. (2008). microRNA-133a regulates cardiomyocyte proliferation and suppresses smooth muscle gene expression in the heart. *Genes Dev.* 22 3242–3254 10.1101/gad.1738708 19015276 PMC2600761

[B58] Lynn F. C. (2009). Meta-regulation: microRNA regulation of glucose and lipid metabolism. *Trends Endocrinol. Metab.* 20 452–459 10.1016/j.tem.2009.05.007 19800254

[B59] Marcheva B. Ramsey K. M. Buhr E. D. Kobayashi Y. Su H. Ko C. H. (2010). Disruption of the clock components CLOCK and BMAL1 leads to hypoinsulinaemia and diabetes. *Nature* 466 627–631 10.1038/nature09253 20562852 PMC2920067

[B60] Memczak S. Jens M. Elefsinioti A. Torti F. Krueger J. Rybak A. (2013). Circular RNAs are a large class of animal RNAs with regulatory potency. *Nature* 495 333–338 10.1038/nature11928 23446348

[B61] Mikkelsen T. S. Ku M. Jaffe D. B. Issac B. Lieberman E. Giannoukos G. (2007). Genome-wide maps of chromatin state in pluripotent and lineage-committed cells. *Nature* 448 553–560 10.1038/nature06008 17603471 PMC2921165

[B62] Moore M. J. (2005). From birth to death: the complex lives of eukaryotic mRNAs. *Science* 309 1514–1518 10.1126/science.1111443 16141059

[B63] Moran I. Akerman I. van de Bunt M. Xie R. Benazra M. Nammo T. (2012). Human beta cell transcriptome analysis uncovers incRNAs that are tissue-specific, dynamically regulated, and abnormally expressed in type 2 diabetes. *Cell Metab.* 16 435–448 10.1016/j.cmet.2012.08.010 23040067 PMC3475176

[B64] Orom U. A. Derrien T. Beringer M. Gumireddy K. Gardini A. Bussotti G. (2010). Long noncoding RNAs with enhancer-like function in human cells. *Cell* 143 46–58 10.1016/j.cell.2010.09.001 20887892 PMC4108080

[B65] Orom U. A. Shiekhattar R. (2013). Long noncoding RNAs usher in a new era in the biology of enhancers. *Cell* 154 1190–1193 10.1016/j.cell.2013.08.028 24034243 PMC4108076

[B66] Pachnis V. Brannan C. I. Tilghman S. M. (1988). The structure and expression of a novel gene activated in early mouse embryogenesis. *EMBO J.* 7 673–681 3396539 10.1002/j.1460-2075.1988.tb02862.x PMC454372

[B67] Penny G. D. Kay G. F. Sheardown S. A. Rastan S. Brockdorff N. (1996). Requirement for Xist in X chromosome inactivation. *Nature* 379 131–137 10.1038/379131a0 8538762

[B68] Poliseno L. Salmena L. Zhang J. Carver B. Haveman W. J. Pandolfi P. P. (2010). A coding-independent function of gene and pseudogene mRNAs regulates tumour biology. *Nature* 465 1033–1038 10.1038/nature09144 20577206 PMC3206313

[B69] Powell W. T. Coulson R. L. Crary F. K. Wong S. S. Ach R. A. Tsang P. (2013). A Prader–Willi locus lncRNA cloud modulates diurnal genes and energy expenditure. *Hum. Mol. Genet.* 22 4318–4328 10.1093/hmg/ddt281 23771028 PMC3792690

[B70] Ramachandran S. Palanisamy V. (2012). Horizontal transfer of RNAs: exosomes as mediators of intercellular communication. *Wiley Interdiscip. Rev. RNA* 3 286–293 10.1002/wrna.115 22012863 PMC3263325

[B71] Rapicavoli N. A. Qu K. Zhang J. Mikhail M. Laberge R. M. Chang H. Y. (2013). A mammalian pseudogene lncRNA at the interface of inflammation and anti-inflammatory therapeutics. *eLife* 2 e00762 10.7554/eLife.00762 PMC3721235 23898399

[B72] Rinn J. L. Kertesz M. Wang J. K. Squazzo S. L. Xu X. Brugmann S. A. (2007). Functional demarcation of active and silent chromatin domains in human HOX loci by noncoding RNAs. *Cell* 129 1311–1323 10.1016/j.cell.2007.05.022 17604720 PMC2084369

[B73] Rottiers V. Naar A. M. (2012). MicroRNAs in metabolism and metabolic disorders. *Nat. Rev. Mol. Cell Biol.* 13 239–250 10.1038/nrm3313 22436747 PMC4021399

[B74] Salmena L. Poliseno L. Tay Y. Kats L. Pandolfi P. P. (2011). A ceRNA hypothesis: the Rosetta Stone of a hidden RNA language? *Cell* 146 353–358 10.1016/j.cell.2011.07.014 21802130 PMC3235919

[B75] Saltiel A. R. Kahn C. R. (2001). Insulin signalling and the regulation of glucose and lipid metabolism. *Nature* 414 799–806 10.1038/414799a 11742412

[B76] Sauvageau M. Goff L. A. Lodato S. Bonev B. Groff A. F. Gerhardinger C. (2013). Multiple knockout mouse models reveal lincRNAs are required for life and brain development. *eLife* 2 e01749 10.7554/eLife.01749 PMC3874104 24381249

[B77] Spitale R. C. Tsai M. C. Chang H. Y. (2011). RNA templating the epigenome: long noncoding RNAs as molecular scaffolds. *Epigenetics* 6 539–543 10.4161/epi.6.5.15221 21393997 PMC3230545

[B78] Stolc V. Gauhar Z. Mason C. Halasz G. van Batenburg M. F. Rifkin S. A. (2004). A gene expression map for the euchromatic genome of *Drosophila melanogaster*. *Science* 306 655–660 10.1126/science.1101312 15499012

[B79] Stoltenburg R. Reinemann C. Strehlitz B. (2007). SELEX–a (r)evolutionary method to generate high-affinity nucleic acid ligands. *Biomol. Eng.* 24 381–403 10.1016/j.bioeng.2007.06.001 17627883

[B80] Sun L. Goff L. A. Trapnell C. Alexander R. Lo K. A. Hacisuleyman E. (2013). Long noncoding RNAs regulate adipogenesis. *Proc. Natl. Acad. Sci. U.S.A.* 110 3387–3392 10.1073/pnas.1222643110 23401553 PMC3587215

[B81] Tay Y. Kats L. Salmena L. Weiss D. Tan S. M. Ala U. (2011). Coding-independent regulation of the tumor suppressor PTEN by competing endogenous mRNAs. *Cell* 147 344–357 10.1016/j.cell.2011.09.029 22000013 PMC3235920

[B82] Thomas D. J. Rosenbloom K. R. Clawson H. Hinrichs A. S. Trumbower H. Raney B. J. (2007). The ENCODE Project at UC Santa Cruz. *Nucleic Acids Res.* 35 D663–D667 10.1093/nar/gkl1017 17166863 PMC1781110

[B83] Trajkovski M. Hausser J. Soutschek J. Bhat B. Akin A. Zavolan M. (2011). MicroRNAs 103 and 107 regulate insulin sensitivity. *Nature* 474 649–653 10.1038/nature10112 21654750

[B84] Tripathi V. Ellis J. D. Shen Z. Song D. Y. Pan Q. Watt A. T. (2010). The nuclear-retained noncoding RNA MALAT1 regulates alternative splicing by modulating SR splicing factor phosphorylation. *Mol. Cell* 39 925–938 10.1016/j.molcel.2010.08.011 20797886 PMC4158944

[B85] van Bakel H. Nislow C. Blencowe B. J. Hughes T. R. (2010). Most ``dark matter'' transcripts are associated with known genes. *PLoS Biol.* 8:e1000371 10.1371/journal.pbio.1000371 PMC2872640 20502517

[B86] Vickers K. C. Palmisano B. T. Shoucri B. M. Shamburek R. D. Remaley A. T. (2011). MicroRNAs are transported in plasma and delivered to recipient cells by high-density lipoproteins. *Nat. Cell Biol.* 13 423–433 10.1038/Ncb2210 21423178 PMC3074610

[B87] Vollmers C. Schmitz R. J. Nathanson J. Yeo G. Ecker J. R. Panda S. (2012). Circadian oscillations of protein-coding and regulatory RNAs in a highly dynamic mammalian liver epigenome. *Cell Metab.* 16 833–845 10.1016/j.cmet.2012.11.004 23217262 PMC3541940

[B88] Wang J. Gong C. Maquat L. E. (2013). Control of myogenesis by rodent SINE-containing lncRNAs. *Genes Dev.* 27 793–804 10.1101/gad.212639.112 23558772 PMC3639419

[B89] Wang K. C. Yang Y. W. Liu B. Sanyal A. Corces-Zimmerman R. Chen Y. (2011). A long noncoding RNA maintains active chromatin to coordinate homeotic gene expression. *Nature* 472 120–124 10.1038/nature09819 21423168 PMC3670758

[B90] Wheeler T. M. Leger A. J. Pandey S. K. MacLeod A. R. Nakamori M. Cheng S. H. (2012). Targeting nuclear RNA for *in vivo* correction of myotonic dystrophy. *Nature* 488 111–115 10.1038/nature11362 22859208 PMC4221572

[B91] Xu B. Gerin I. Miao H. Vu-Phan D. Johnson C. N. Xu R. (2010). Multiple roles for the non-coding RNA SRA in regulation of adipogenesis and insulin sensitivity. *PLoS ONE* 5:e14199 10.1371/journal.pone.0014199 PMC2996286 21152033

[B92] Yang F. Zhang H. Mei Y. Wu M. (2014). Reciprocal regulation of HIF-1alpha and LincRNA-p21 modulates the Warburg effect. *Mol. Cell* 53 88–100 10.1016/j.molcel.2013.11.004 24316222

[B93] Yang L. Lin C. Jin C. Yang J. C. Tanasa B. Li W. (2013). lncRNA-dependent mechanisms of androgen-receptor-regulated gene activation programs. *Nature* 500 598–602 10.1038/nature12451 23945587 PMC4034386

[B94] Yap K. L. Li S. Muñoz-Cabello A. M. Raguz S. Zeng L. Mujtaba S. (2010). Molecular interplay of the noncoding RNA ANRIL and methylated histone H3 lysine 27 by polycomb CBX7 in transcriptional silencing of INK4a. *Mol. Cell* 38 662–674 10.1016/j.molcel.2010.03.021 20541999 PMC2886305

[B95] Zhou B. Li C. Qi W. Zhang Y. Zhang F. Wu J. X. (2012). Downregulation of miR-181a upregulates sirtuin-1 (SIRT1) and improves hepatic insulin sensitivity. *Diabetologia* 55 2032–2043 10.1007/s00125-012-2539-8 22476949

